# Focus Groups in Small Communities

**Published:** 2010-04-15

**Authors:** Nicolette I. Teufel-Shone, Sheralyn Williams

**Affiliations:** Mel and Enid Zuckerman College of Public Health; Mel and Enid Zuckerman College of Public Health, University of Arizona, Tucson, Arizona

## Abstract

Qualitative research methods have gained increasing acceptance as valuable tools for gathering information on attitudes, beliefs, and sociocultural factors that influence health behaviors. Conducting focus groups is a commonly used qualitative method. Existing guidelines for conducting focus groups do not address the challenges presented by the social familiarity of small communities and do not highlight the advantages of using the technique as part of a community-based participatory research (CBPR) effort. In small communities, researchers must consider characteristics of the facilitator and recorder, recruitment strategies, the importance of stressing confidentiality even when discussing seemingly nonsensitive topics, and the effect of disseminating results. Addressing these elements as part of a CBPR approach is advantageous because community partners know the ways in which the community talks about an issue and understand the subtle social impact of asking, answering, and interpreting locally specific questions.

## Introduction

In public heath, researchers frequently use focus groups to document people's range of beliefs, opinions, and experiences relative to a specific topic (1-3). Protocols describe the typical focus group as 6 to 10 people, but some guidelines and research reports note that groups as small as 4 and as large as 12 can be productive (1,4,5). The standard recommendation for group formation is to select participants who are reasonably homogeneous and unfamiliar with not only each other but also the facilitator (4,5). This recommendation makes the use of focus groups in small, socially connected communities challenging because potential participants may be hesitant to disclose their experiences with a "stranger" (ie, the facilitator) and are likely to have regular contact with other group participants (3,6,7).

Our experiences implementing focus groups in small communities have led to the adaptation of existing protocols to accommodate the level of familiarity and social dynamics of this social setting. We offer the following recommendations to yield techniques to fit the sociocultural context. These recommendations are best applied as a part of a community-based participatory research (CBPR) approach guided by an investigative team composed of community ("inside") and research ("outside") collaborators (1,5,7). Many of the issues highlighted in this article would be easily resolved in an "insider-outsider" investigator partnership (2,8).

## Focus Group Elements

### Facilitator

In a small community, use of a facilitator who is from the community (an "insider") or not from the community (an "outsider") influences the way information is shared and possibly its content. With an outside facilitator, participants minimize their use of "insider" jargon (ie, local terms for places and agencies) and may elaborate on specific behaviors or conditions participants perceive as unfamiliar to the outside facilitator. This clarity of information is useful in analyzing and disseminating the information for an outside audience. However, for the participants, the time needed to explain the "basics" may detract from discussing more complex issues related to the topic.

Working in CBPR projects, Kieffer et al (1) and Christopher (6) in her work with American Indians and reminiscent of the work of Banner et al indicate that their community partners advocated for community members to be trained to facilitate focus groups. An ideal facilitator should be a trusted community member familiar with the informal and formal power and social structures of the community. This person should not be a formal community leader at the time of conducting the focus groups and should be known as a nonjudgmental listener. Being known as an "ardent adviser" or as strongly opinionated is not desirable for a focus group facilitator. A facilitator should be perceived locally as a moderate. A socially influential facilitator might elicit habitual or even defensive responses from participants and may not invite the reflective, open responses desired in a focus group.

These characteristics are more important if the discussion includes politically, socially, or personally sensitive topics. We recommend investing time to solicit anonymous recommendations from local collaborators. One method we have used successfully is to list the 3 desirable traits discussed above (listener, nonjudgmental, and political/social moderate) and ask several community collaborators to identify 3 local people who best exemplify these traits. In our experience, collaborators identify 1 to 3 of the same people.

Focus groups can best discuss some topics if the facilitator has the same ethnic background as the participants if a fairly ethnically homogenous group is recruited (1). The importance of this characteristic should be discussed and a decision made by the investigative CBPR team. In our experience, having an outside facilitator of the same ethnicity as the focus group participants was met with mixed response and even concerns of internalized racism: "She looks like us, but she still doesn't know us" and "She thinks we are uneducated. You can tell. Her voice was flat and she never laughed." This reaction is noted by Christopher (6) and reminiscent of the work of Banner et al (9) with Native Hawaiians, who reacted negatively to surveyors who used a standard neutral voice tone and showed little response to interviewees' answers.

In keeping with the principles of CBPR, the facilitator(s) should be engaged in discussions of the direction and wording of focus group questions, in the development of recruitment strategies to fit local behaviors and networks, in data analysis to align the interpretation with the local context, and in dissemination to ensure that outcomes are understandable and applicable to the community. As experts in local behaviors, community members can best predict if potential participants will take part in focus groups, for example,  in the evening or on a Saturday morning and if transportation or child care are potential barriers and should be provided. If facilitators are paid an hourly rate, the amount should reflect the local assurance and legitimacy the community facilitator is bringing to the process.

Christopher (6) provides a guide to using a CBPR approach to train community interviewers. Although these recommendations are drawn from work with an American Indian community, this guide is useful in training community members from other socially and culturally connected settings. A key concept discussed is incorporating local ways of interacting, questioning, and probing to yield a comfortable and natural process.

### Recording and recorder

In a small community, focus group participants may be concerned that their voice will be recognized if sessions are audiotaped. This concern may be present regardless of the topic discussed. In our experience, focus group participants discussing local recreational resources elected not to participate if the sessions were audiotaped. This example highlights the importance of using a CBPR approach; sensitivity is defined by the social and cultural context.

When audiotaping was a deterrent, we used scribes to manually document discussions. We avoided using a laptop computer because the sound of notetaking was distracting to some participants. Two scribes or notetakers were used to provide a complete account of the responses. Notetakers worked together to draw a duplicate map on their writing pad illustrating the seating arrangement of the participants, the facilitator, and the notetakers, and to assign a position number to each participant. The participant's position number was used to document his or her comments and nonverbal gestures that might add meaning to the discussion (1,2). Both notetakers worked to record all statements. An alternative approach — assigning certain participants to each notetaker — distributes the flow of the discussion, but notetakers become disengaged and do not produce coherent notes. An ideal arrangement in a CBPR setting would be to have an inside investigator such as a trained community member conduct the focus group and to have 1 notetaker be a community member and 1 be an outside investigator. In this configuration, the community or inside notetaker would understand any local jargon or references and the outside notetaker would pick up local statements that may seem commonplace to the inside notetaker but are valuable for further investigation.

Immediately or within a day of the focus group, the notetakers should meet to synthesize their notes and produce a collective and single document of the discussion including the seating map and start and end times.

### Questions

In small communities, researchers should consider the local culture to ensure that the intent of the question is clear. For example, in the aforementioned focus groups about recreational facilities, the investigative team modified questions extracted from a national program and used local terms. The national question, "Do you use local outside recreational resources such as walking paths and sports courts?" yielded responses of "we don't have those resources in our community." The phrase implies the formal development of a path designed specifically for recreational walking. Yet the community has several footpaths worn down by community members walking to and from church, the store, or the post office. The question was reworded as "Do you use the Church Hill loop or the store path for exercise?"

Guides to focus group question development generally advise facilitators to ask participants to speak from experience and encourage response in the first person. Krueger and Casey (4) offer a generally useful strategy of asking participants to complete a statement (eg, "When I found out my cholesterol was high, I felt . . ."). In small communities, this approach may ask the participant to reveal too much personal information. In our experience, the recommended first-person approach yielded silence or terse responses. We have found that modifying the question to allow for a third-person response yielded a less guarded response: "When a person finds that his/her cholesterol is high, he/she might feel . . . ." Rephrasing the statement does solicit a different response. Asking participants to project how someone might feel does not solicit information about a personal experience, but respondents often do speak from personal experience. If the intent of the investigation is to gather personal stories and experiences in the first person, in a small community these data might be best collected in face-to-face interviews rather than in focus groups.

### Setting

In all communities, the setting can affect participants' comfort level in discussing particular topics (4,7). For example, participants may give guarded responses to questions about teenage pregnancy if the setting is a church meeting room or school. In a small community with limited meeting spaces, outside investigators should be aware of recent local events that temporarily affect the aura or atmosphere of a particular setting. For example, funeral services and wakes are sometimes held in local gymnasiums and community centers, and scheduling a focus group in that building in the days following may affect community members' willingness to participate or talk about certain topics. Death and other seminal events affect most everyone in a small community. The best strategy, especially if no other site is available, is to postpone the focus group for a week or so to get more accurate responses.

### Timing

Researchers should schedule focus groups to match the availability of the target group; for example, evenings or weekends are best if potential participants work a standard 8-hour day, and mid-morning may work for older adults who congregate for lunch at a senior center.

Other circumstances to consider are local or cultural seasonal events or rituals that would influence community members' willingness and ability to participate. Not scheduling focus groups around recognized religious or national holidays seems obvious to many investigators, but other conflicting events may be less apparent. An outside investigator who attempted to recruit a focus group in an American Indian community around a multiday, annual culture-specific event was frustrated by her lack of success. She rescheduled her sessions after a community member explained the conflict to her. This situation reinforces the importance of using a CBPR approach; local experts should contribute to a discussion of scheduling and site selection.

### Recruitment

A CBPR approach is invaluable to the development of appropriate recruitment strategies (6). In small communities, word of mouth can be effective especially if a local contact or referral person is available and easily accessible. Many small communities have weekly, monthly, or bimonthly newsletters or newspapers. The deadline for submitting notices may be as much as a week before the distribution date. Missing the deadline for a monthly publication can significantly delay the scheduling of a focus group.

Other recruitment avenues include announcements on local radio stations and posting notices in local high-traffic areas. In our experience, announcements broadcast from nonlocal radio stations are not effective in reaching eligible participants. If a local station is available, get information on formats, available time slots, costs, protocols for submission and review, and other requirements. Community collaborators should take the lead in developing the announcement to ensure that the message is easily understood and explains community benefits and participant compensation. If the local radio announcer will not be reading the announcement, the local reader should be a well-respected community member. Be prepared to compensate people who do not work for the project for their time and willingness to lend their reputation to validate the focus group process.

Posted notices are pervasive in small communities. Recruitment materials need to be visually appealing  and eye-catching to stand out. We recommend using graphics and color print or paper. Consider using a short phrase in large print that capitalizes on a popular local saying or mass media advertisement campaign (eg, "GOT THOUGHTS?" or "WHAT DO YOU THINK?") to catch the eye of people passing by a community bulletin board covered with local flyers. The notice should include eligibility criteria and a local contact person, both telephone numbers and physical location to accommodate those lacking telephone access.

### Assurance of confidentiality

A challenge in focus groups administered in a socially connected small community is assuring participants that their statements will be confidential. Researchers should address confidentiality during the training of facilitators (6) and discuss it at the onset of the focus group. Remind participants that they, the facilitators, and notetakers are entrusted with the information being shared in the focus group.

To reinforce the credibility of the focus group process, facilitators should explain the following:

1. The intent of the focus group is to understand local thoughts and opinions to inform and improve an ongoing service or to propose a new intervention to fit the needs of the community.
2. A summary of the focus group will be shared with the local community and possibly the larger public heath and scientific communities.
3. Names of participants will not be revealed or linked to any particular statements.

Although these statements are written on the subject consent or disclaimer form signed by the participants, repeating the concepts reinforces the significance of the activity. In addition, the local facilitator and notetakers must be trustworthy to assure confidentiality.

## Analysis

Using a CBPR approach in the analysis of focus group responses from a small community is key to understanding the local subculture or context that influences participants' word choice, internal consistency or opinion shifts, frequency and intensity of comments, and specificity of responses (4). We recommend a systematic means of engaging the insider and outsider perspective of the CBPR investigative team in the analysis process. The multi-investigator consensus method offers a guide to identifying the patterns and themes within participants' statements (10). This method is based on Patton's (11) description of content analysis of searching text, such as focus group notes, for recurring words, concepts, or ideas. Three investigators independently identify these recurring words or ideas to reveal a pattern. A pattern is a descriptive finding that summarizes the recurring statements (eg, "Most parents feel undermined by the number of soft drink advertisements that target youth" or "Parents report that their health messages are overpowered by TV advertisements"). The 3 investigators convene to share their individually identified patterns and to discuss different interpretations. They then consider the patterns collectively, reaching consensus to develop a theme. A theme takes a more topical form: "Parents feel disempowered."

Community engagement is needed to ensure that data interpretation occurs with a relevant sociocultural context, to yield an appropriate data dissemination plan, and to incorporate findings into future health promotion activities.

## Dissemination

Dissemination of results and feedback to the community is an important aspect of the focus group process (7). In a small, cohesive community, community members will remember when the groups were conducted, will probably know who participated, and will be concerned about what the process will reveal about their community. Results from small samples should be generalized and should not include quotes that allow direct association between a specific statement and a particular participant. Community or inside investigators can determine which direct quotes might be suitable for public dissemination, that is, they can discern if a statement could have made by many community members or if everyone will know who said it. This recommendation for the selection of representative statements adheres to standard analytical procedures, which highlight the discovery of normative behaviors and opinions, not outliers. Researchers should complete local dissemination in a timely manner. Consider multiple formats; all interested community members may not be able to attend a single public presentation. Possible avenues include public presentation led by the community investigators and supported by the outsider investigators, handouts of a PowerPoint presentation or a 1-page highlights sheet, a 1- to 2-page article in the local paper, or a radio narrative.

## Conclusion

Focus groups are a useful tool for providing insight into people's experiences, beliefs, and opinions. Qualitative data are needed to identify barriers and promoters of particular health behaviors, guide the development of socially and culturally relevant intervention strategies, and assess the subtle and perhaps normative impact of an intervention. Focus groups rely on everyday ways of communicating and do not rely on literacy or familiarity with specific terminology or technology. Focus groups' reliance on verbal communication requires that investigators be alert to factors that influence local styles of communication. In a small community, most focus group participants will know each other. The lack of anonymity may suppress the openness that yields the content-rich data desired from a focus group. Standard focus groups designed for use in the indifferent or loose social networks of a large community or city can be adapted for use in a small community setting where social familiarity and concerns of confidentiality can affect candidness. Researchers should consider all aspects of the focus group elements when adapting the focus group process. Using a CBPR approach ensures the engagement of community members who can collaborate on the adaptive process and can provide valuable insiders' perspectives on the documentation, analysis, and dissemination of research outcomes. Focus groups are an invaluable research method. The method is enhanced by adapting to the sociocultural setting of the data collection site.
